# Selection and validation of suitable reference genes for qRT-PCR analysis in pear leaf tissues under distinct training systems

**DOI:** 10.1371/journal.pone.0202472

**Published:** 2018-08-23

**Authors:** Zheng Liu, Kexin Cheng, Zhongqi Qin, Tao Wu, Xianming Li, Junfan Tu, Fuchen Yang, Hongyan Zhu, Li Yang

**Affiliations:** 1 Research Institute of Fruit and Tea, Hubei Academy of Agricultural Sciences, Wuhan, Hubei, China; 2 School of Life Sciences, Wuhan University, Wuhan, Hubei, China; Nazarbayev University, KAZAKHSTAN

## Abstract

Training systems generally alter tree architecture, which modulates light microclimate within the canopy, for the purpose of improving photosynthetic efficiency and fruit quality. Gene expression quantification is one of the most important methods for exploring the molecular mechanisms underlying the influence of training systems on pear photosynthesis, and suitable reference genes for gene expression normalization are a prerequisite for this method. In this study, the expression stability of nine common and four novel candidate genes were evaluated in 14 different pear leaf samples in two training systems, including those at four developmental stages (training_period) and from different parts of the trees (training_space), using two distinct algorithms, geNorm and NormFinder. Our results revealed that *SKD1* (*Suppressor of K*^*+*^ *Transport Growth Defect1*)/ *YLS8* (*Yellow Leaf Specific 8*) and *ARM* (*Armadillo*) were the most stable single reference genes for the ‘training_period’ and ‘training_space’ subsets, respectively, although these single genes were not as stable as the optimal pairs of reference genes, *SKD1*+*YLS8* and *ARM*+*YLS8*, respectively. Furthermore, the expression levels of the *PpsAPX* (*Ascorbate peroxidase*) gene showed that the arbitrary use of reference genes without previous testing could lead to misinterpretation of data. This work constitutes the first systematic analysis regarding the selection of superior reference genes in training system studies, facilitating the elucidation of gene function in pear and providing valuable information for similar studies in other higher plants.

## Introduction

Pear is one of the most economically important deciduous fruit trees globally, and training is a vital cultivation technique to maintain its optimal fruit quality. A good training system is an important foundation for manipulations of tree planting arrangement and canopy geometry to improve the penetration and distribution of photosynthetically active radiation (*PAR*) for the purpose of optimizing photosynthetic carbon fixation and fruit quality [1]. Canopy structure can alter microclimate conditions (temperature, humidity and other environmental factors) and thereby indirectly affect disease incidence [2,3]. Currently, there are various training systems used for growing pears, such as the spindle system, open vase system, trellis system and so on. However, no one training system is best for all conditions (cultivar, rootstock, climate or economic circumstance) [4]. Therefore, understanding and utilizing the advantages of various training systems could be beneficial for pear growth and productivity.

In China, free systems, such as spindle and open vase, are widely employed in most pear orchards because of their low costs and high early yields. However, fruit may bruise easily and reduce marketability when the branches are not firmly attached to supports [5]. At the intra-tree scale of traditional training systems, fruit quality may show a large variability in response to architectural position, since uneven distribution of light within the canopy is associated with shade [6,7]. The flat-type trellis system has been widely utilized for pear cultivation in Japan. Recently, this system has attracted interest and gradually developed in China. The training system is based on the removal of water shoots above the trunk or primary branches and tying the lateral shoots with string to the trellis. This arrangement allows trees to receive sunlight uniformly and achieve uniform fruiting and fruit quality, not to mention lower labor costs and its capability to protect fruit trees from wind [5]. In addition, this system increases the shoot angle from the vertical to the horizontal position, which can promote flower bud formation and fruit set [8]. Although the flat-type trellis system favors low-density plantings, thereby gaining lower early productivity, the yield increases when trees grow into adults with more shoots.

Branch or shoot bending is one of the most typical structural characteristics of the flat-type trellis system. Such mechanical stress may regulate hormone pathway genes, which might alter endogenous hormone levels and signaling sensitivity, thus promoting flower induction and increasing fruit production [8]. In ‘Kiyo’ Japanese plum, bent shoots in a joint tree system and the horizontal shoots of stand-alone trees showed lower levels of *PslGA3ox* transcript, which encodes a key enzyme involved in GA biosynthesis and may promote flower induction, than the upright shoots of stand-alone trees [9]. In pear, the expression levels of ripening-related genes (*EIN4*, *ERS1*, *ETR1*, *ETR2*, *PG*, *ACO1*, *APRT*, *Def*, *MnSOD*, *GAL* and *PGM*) were higher in a V-shaped system or the bottom part of the canopy that had less ripe fruit, which might indicate competence to initiate the ripening process [10]. The trellis system may improve light penetration and distribution in the orchard by training technology that decreases within-shoot shading, which appears to be crucial for fruit quality. However, very few studies offer a direct comparison of pear training systems, and the underlying molecular mechanisms explaining the contribution of efficient training system remain unknown [5,11,12]. Therefore, by comparing the potential photosynthesis of the classical central leader training system and trellis system and revealing the functions of biomolecules in nutrient contribution, we could better understand the mechanisms by which shoot reorientation in the trellis system promotes fruit quality. Gene expression analysis is an important research method to gain insight into the putative functions of specific candidate genes involved in complex regulatory relationships. Among the widely used methods, quantitative real-time reverse transcription PCR (qRT-PCR) is a sensitive and reproducible technique with a broad quantification range for measuring gene expression [13–16]. This technique has been recently used to quantify changes in gene expression profiles in response to developmental transitions (anthocyanin biosynthesis, sclereid formation, fruit development) and environmental changes (low temperature, dehydration stress, biotic stress) in pear [17–22]. Nevertheless, to accurately quantify gene expression by qRT-PCR, it is necessary to normalize the expression levels of the target genes to the expression levels of optimal reference genes, which are expected to exhibit only minor differences in their expression across various tissue/organ types, developmental stages and experimental conditions [23]. However, none of the reference genes discovered thus far is consistently expressed under diverse experimental situations [24–27]. Previously, a few so-called housekeeping genes, such as *glyceraldehyde-3-phosphate dehydrogenase* (*GAPDH*), *elongation factor 1a* (*EF1a*) and *tubulin* (*TUB*), have been used extensively as internal controls for gene expression analysis in pear without systematic evaluation of their expression stability in a preliminary experiment [22,28,29]. There is evidence that the transcript levels of housekeeping genes may vary considerably under some experimental conditions [30]. Currently, two Excel-based statistical algorithms, geNorm and NormFinder, are widely recognized for selecting suitable reference genes in various species [31–36]. Based on these algorithms, only four studies relying on qRT-PCR analysis in pear have evaluated the stability of candidate reference genes, mainly in a few test conditions such as various tissues or exposure to some abiotic stresses [37–40]. However, no systematic study has been carried out for the selection of reference genes in distinct training systems.

In this study, we used qRT-PCR to examine variations in the expression of thirteen candidate reference genes, including nine common candidate reference genes (*Actin2*, *EF1a*, *GAPC*, *Histone H3*, *SAND*, *TIP41-like*, *TUB7*, *UBQ5* and *YLS8*), which were previously shown to have highly stable expression levels in pear in other experimental conditions [37–39], and four novel reference genes (*ARM*, *MYB10*, *SKD1* and *SRP34A*) retrieved from our transcriptome data of fruit tissues between the flat-type trellis system and traditional spindle system. Our data provide optimal single or multiple reference gene(s) that will enable more accurate and reliable qRT-PCR normalization for gene expression studies in pear under distinct training systems and canopy positions. These results provide a useful basis for future gene expression analysis in studies of pear training system.

## Material and methods

### Ethics statement

This study was approved by the Wu Chang Sand Pear Garden National Fruit-tree Germplasm Resource (WCSPGNFGR), Wuhan, China. No specific permissions were required for the described field studies, as the pear germplasm resource were managed through Research Institute of Fruit and Tea. The collection location is not privately owned or protected area, and the field studies did not involve endangered or protected species.

### Plant materials

‘Wonhwang’ pear (*Pyrus pyrifolia* Nakai cv. Wonhwang) leaf samples of distinct training systems (flat-type trellis system and spindle system) were collected from WCSPGNFGR. A flat-type trellis system was developed by our research team and referred to as Double Primary Branches Along the Row (DPBAR) (Figures a, b, and c in S1 Fig). The tree canopy is supported on two horizontal steel wires that are stretched along the row at heights of 1.3 m and 1.7 m. The two primary branches are bent in opposite directions along the row and hung naturally on the top wires. In the traditional spindle system, the tree has a central and vertical leader with a few horizontal branches, which are shorter at the tree top than at the bottom (Figure d in S1 Fig). Approximately twenty fully expanded sixth leaves in all directions were collected from the middle of May to August, which covers the full transition from fruit set to harvest maturity. These eight leaf samples were designated as the subset ‘training_period’, representing the leaf samples at four development stages in two distinct training systems. To inspect the stability of candidate reference genes over a wider spectrum, another six fully expanded leaf samples were collected in July under the two distinct training systems and designated as the second subset ‘training_space’, including those from the exterior, central and interior parts of the trees in the two distinct training systems (Figure e in S1 Fig). All samples mentioned above were collected from three independent trees as three biological replicates, immediately frozen in liquid nitrogen and stored at -80°C until total RNA isolation.

### Total RNA isolation, quality control and cDNA synthesis

Total RNA was extracted from the frozen leaves using RNAprep Pure Plant Kit (Polysaccharides & Polyphenolics-rich) (Tiangen, China) following the manufacturer’s instructions. RNA concentration of each sample was determined using a NanoPhotometer^TM^ spectrophotometer (IMPLEN, Germany). The OD_260_/OD_280_ absorption ratio (1.9 and 2.1) and OD_260_/OD_230_ (>2.0) were used to determine the quality and purity of RNA samples. Total RNA integrity was also verified by Agilent 2100 bioanalyzer (Figure a in S2 Fig). First-strand cDNA was synthesized from 1 μg of total RNA using RevertAid^TM^ First Strand cDNA Synthesis Kit (Fermentas, USA). cDNA was stored at -20°C for future use.

### Candidate reference gene selection and primer design

The nine candidate reference genes (*Actin2*, *EF1a*, *GAPC*, *Histone H3*, *SAND*, *TIP41-like*, *TUB7*, *UBQ5* and *YLS8*) were selected based on their common usage as reference genes in previous pear studies, where they showed stable expression levels in other tested experimental conditions [37–39]. For all candidates mentioned above, the primers from previous studies were used. These genes represent several functional classes to minimize the possibility of co-regulation (Table 1). In addition, we included four novel candidates, i.e., *ARM* (*Armadillo*; accession no. MG029158), *MYB10* (Myeloblastosis 10; accession no. MG029159), *SKD1* (*Suppressor of K*^*+*^ *Transport Growth Defect1*; accession no. MG029160) and *SRP34A* (Serine/Arginine-Rich Protein Splicing Factor 34A, accession no. MG029161), which were identified from transcriptome datasets of ‘Wonhwang’ pear fruit samples during the maturation period in the same two training systems and found to have approximately even expression levels among libraries (S1 Table). However, the expression stability of these candidates should be still tested, since there is no additional evidence to verify that the four genes may be equally expressed in leaves under distinct training systems. Primers were designed by Primer 5.0 software using the default parameters (Table 1). To confirm the sequences of the amplicons, the PCR products were analyzed on 1.2% agarose gel and then sequenced. No-template control and no-reverse-transcriptase controls were performed as well (Figure b in S2 Fig). The amplicons of the 13 candidate reference genes were used to obtain homologous genes through a BLASTN search against the *Arabidopsis thaliana* database (The Arabidopsis Information Resource, http://www.arabidopsis.org/).

**Table 1:** Genes, primers and amplicon characteristics.

*Gene* *abbreviation*	*A*. *thaliana* *ortholog locus*	*Gene function*	*Similarity* *(e-value)*	*Primer sequences (forward/reverse)*	*Length* *(bp)*	*Efficiency*	R^2^
*Actin2*	AT3G18780	Cytoskeletal structural protein	2e-31	F: CTCCCAGGGCTGTGTTTCCTA	173	2.03 ±0.038	0.999
				R: CTCCATGTCATCCCAGTTGCT			
*ARM*	AT3G08947	NA	0.28	F: CAAGGGCATTCTTTCGG	101	1.98 ±0.057	0.994
				R: CAGGGACATCATTAGGAACAT			
*EF1a*	AT1G07930	Translation initiation factor 1a	1e-35	F: GGTGTGAAGCAGATGATTTG	167	2.00±0.033	0.999
				R: TCACCCTCAAACCCAGATAT			
*GAPC*	AT3G04120	Carbohydrate metabolism	4e-22	F: TGGTGTGAACGAGAAGGAAT	120	2.06±0.029	0.996
				R: CCCTCAACAATCCCAAACC			
*Histone H3*	AT4G40040	Involved in structure of chromatin	4e-32	F: GTCAAGAAGCCCCACAGATAC	153	2.00 ±0.034	0.997
				R: CTGGAAACGCAGATCAGTCTTG			
*MYB10*	AT3G12820	NA	0.23	F: GGGAACAACAGCAAACG	87	1.93 ±0.096	0.995
				R: CTCCGAAGCACTACCATTA			
*SAND*	AT2G28390	Hypothetical proteins	5e-10	F: CCCAGGACTTTGAGCTTTATGC	145	2.09 ±0.033	0.996
				R: TATCACCATGAAAAGGGGCTTG			
*SKD1*	AT2G27600	Maintenance of the large central vacuole	7e-15	F: CTTCCGCCTCCTATCAC	127	2.04 ±0.014	0.998
				R: TTCATCACCCTTCCTCT			
*SRP34A*	AT1G31230	NA	0.1	F: CGGTAGTGCCTGATTCTC	139	2.10± 0.084	0.996
				R: CATACTCGCCATAACAAAG			
*TIP41-like*	AT4G02660	NA	1.3	F: ATCCAAGCATCATCAGCCAAAG	113	2.11 ±0.048	0.996
				R: GGAACAATAACTCTTGCAGGGAGA			
*TUB7*	AT2G29550	Microtubules of the eukaryotic cytoskeleton	3e-33	F: TGGGCTTTGCTCCTCTTAC	171	2.03 ±0.085	0.996
				R: CCTTCGTGCTCATCTTACC			
*UBQ5*	AT3G62250	Constituents of mature ribosomes	4e-08	F: ACCCTCGCCGACTACAAC	199	2.05 ±0.030	0.999
				R: ACTCCTTCCGCAGCCTCT			
*YLS8*	AT5G08290	Mitosis protein	2e-21	F: TGAGGTGCTGGCTTCTGT	119	2.09 ±0.047	0.996
				R: TGACCGTTGATGGATCGTA			

NA indicates that the gene’s function could not be predicted because of low similarity with *A*. *thaliana* orthologs.

The accession numbers of the four novel candidates are MG029158 (*ARM*), MG029159 (*MYB10*), MG029160 (*SKD1*) and MG029160 (*SRP34A*).

### Quantitative real-time reverse transcription PCR (qRT-PCR) and PCR efficiency determination

qRT-PCR was performed utilizing SYBR Green detection chemistry on an ABI 7500 Real Time System (Applied Biosystems, USA). The qRT-PCR cocktail contained 0.5 μl of cDNA, 0.5 μl each of specific sense and anti-sense primers and 5 μl of 2× SYBR GREEN PCR Master Mix (Applied Biosystems, USA) in a final reaction volume of 10 μl for each well. The PCR amplification program was as follows: an initial denaturation step at 95°C for 3 min, followed by 40 amplification cycles of 30 s at 95°C, and 60°C for 1 min. Three biological replicates for each sample and four technical replicates of each biological replicate were analyzed by qRT-PCR. Each run was completed with a melting curve analysis for 13 candidate reference genes to confirm the specificity of amplification. A serial dilution of the total combined cDNA pools was used to obtain standard curves and the corresponding primer amplification efficiency for each candidate reference gene.

### Determination of reference gene expression stability using geNorm and NormFinder

Analysis tools including geNorm (version 3.5) and NormFinder (version 0.953) were used to estimate gene expression stability [31, 32]. Genorm is a Microsoft Excel application that calculates the average stability measure (M) by assessing the pairwise expression ratio of each reference gene against all other tested reference genes. Genes with the lowest M value are considered to be most stably expressed. The pairwise variation V_n_/V_n+1_ between two sequential normalization factors was calculated to determine the optimal number of reference genes required for accurately normalizing qRT-PCR. A threshold value below 0.15 suggests that no additional reference genes are required for normalization. NormFinder uses an ANOVA-based model to estimate both intra- and inter-group variations and provides a direct measure for integrating all consistent results.

### Validation of reference genes

In perennial fruit trees, including pear, complex branching structure and high leaf density also potentially aggravate environmental stress (high temperature or strong light) [41]. Ascorbate peroxidase (APX) was shown to be involved in scavenging H_2_O_2_ under stress conditions. To evaluate the validity of the reference genes, the expression profile of a target gene, *PpsAPX* (*Ascorbate peroxidase*), was detected in the eight samples of the ‘training_period’ subset and normalized separately to the most stable single reference gene, the optimal reference gene pair, two traditional reference genes and two unstable reference genes. For *PpsAPX*, primers were previously designed by Liu et al. [41].

## Results

### RNA intergrity assessment, amplification specificity and PCR efficiency

To verify the RNA integrity, Agilent 2100 bioanalyzer was used to determine RNA quality. The sizes of the RNA bands (28S and 18S) were conformed to their theoretical values (Figure a in S2 Fig). However, an abnormal migration was observed for chloroplast 23S rRNA, since it migrated slightly faster than 18S rRNA, presumably due to secondary structures effects that were not resolved by non-denaturing capillary electrophoresis. The RIN values of all samples were higher than seven, indicating that these RNA starting materials can be used for subsequent experiments. Agarose gel electrophoresis revealed that all primers of the 13 candidate reference genes amplified a single PCR product with the expected size, and then the cloned amplicons were sequenced (S2 Table). No amplification was observed in the negative control reactions with no template or no reverse transcriptase (Figure b in S2 Fig). The specificity of each primer pair was further confirmed by the presence of a single peak in the melting curve analysis (S3 Fig). The PCR amplification efficiency of the thirteen reference genes ranged from 92.86% for *MYB10* to 110.95% for *TIP41-like* with linear regression coefficient R^2^ values being >0.99 (Table 1).

### Expression levels of candidate reference genes

**Fig 1:**
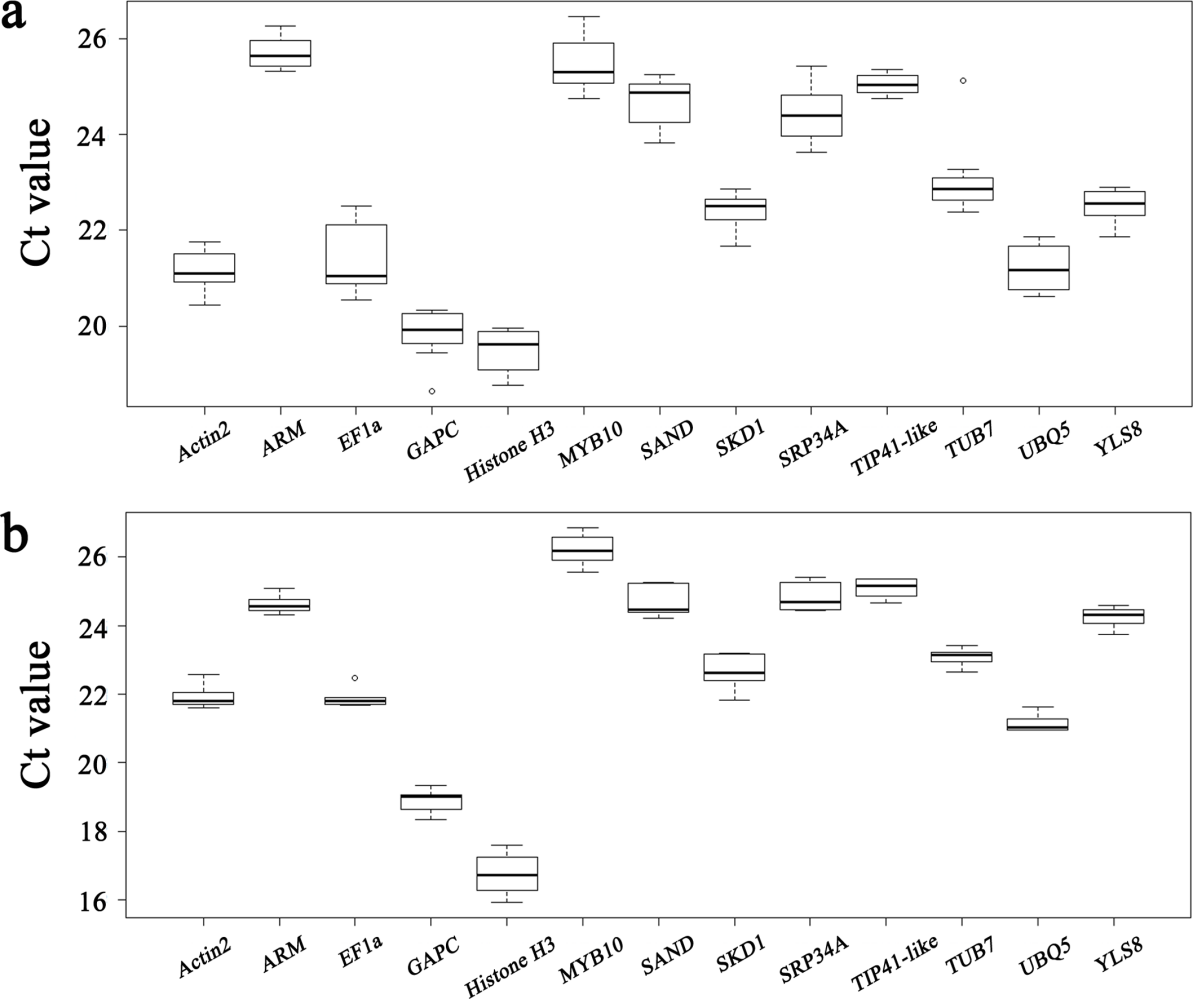
Ct values for 13 candidate reference genes across samples. The Ct values of the candidate reference genes in the ‘training_period’ subset (a) and the ‘training_space’ subset (b). The box represents the 25th and 75th percentiles of the data. A line across the box depicts the median. Whiskers extend to the minimum and maximum values. Circles indicate outliers.

The quantification cycle (Ct) values of all 13 candidate reference genes were variable across the tested samples, indicating that their gene expression levels are affected by developmental stage, spatial distribution and training system (Fig 1). Naturally, the lowest amount of variance is the most favorable.

For leaf tissues at four development stages in two distinct training systems (hereafter referred to as the ‘training_period’ subset), the Ct values of the candidate reference genes ranged from 18.64 to 26.46 (Fig 1A). Among these genes, *Actin2*, *EF1a*, *GAPC*, *Histone H3* and *UBQ5* were transcribed most abundantly, with Ct median values 21.15, 21.39, 19.83, 19.49 and 21.21, respectively, whereas *ARM* presented the lowest average expression level (mean Ct = 25.70). *EF1a* displayed relatively higher variation in the ‘training_period’ subset, with Ct values 20.55–22.50. In contrast, *SKD1* and *YLS8* were expressed rather stably, with a narrow range of Ct values spanning 21.67–22.86 and 21.87–22.90 among samples, respectively.

In the leaf tissues from various parts of the trees in the two distinct training systems (hereafter referred to as the ‘training_space’ subset), the 13 candidate reference genes were also expressed differentially among samples, with Ct values spanning 15.93–26.86 (Fig 1B). *GAPC* and *Histone H3* showed high accumulation, with median Ct values 18.90, and 16.75, respectively, whereas *MYB10* had the lowest average expression level (mean Ct = 26.21). From the Ct value ranges of each candidate reference gene, we could tell that the expression levels of *Actin2*, *ARM* and *UBQ5* were relatively stable among the ‘training_space’ subset tissues, with Ct values spanning 21.61–22.57, 24.32–25.09 and 20.96–21.62, respectively. On the other hand, *Histone H3* was the most variable across the subset samples, with Ct values spanning 15.93–17.59, suggesting that *Histone H3* may not be suitable as a reference gene in these experimental conditions.

### Expression stability assessment of the candidate reference genes by geNorm

**Fig 2:**
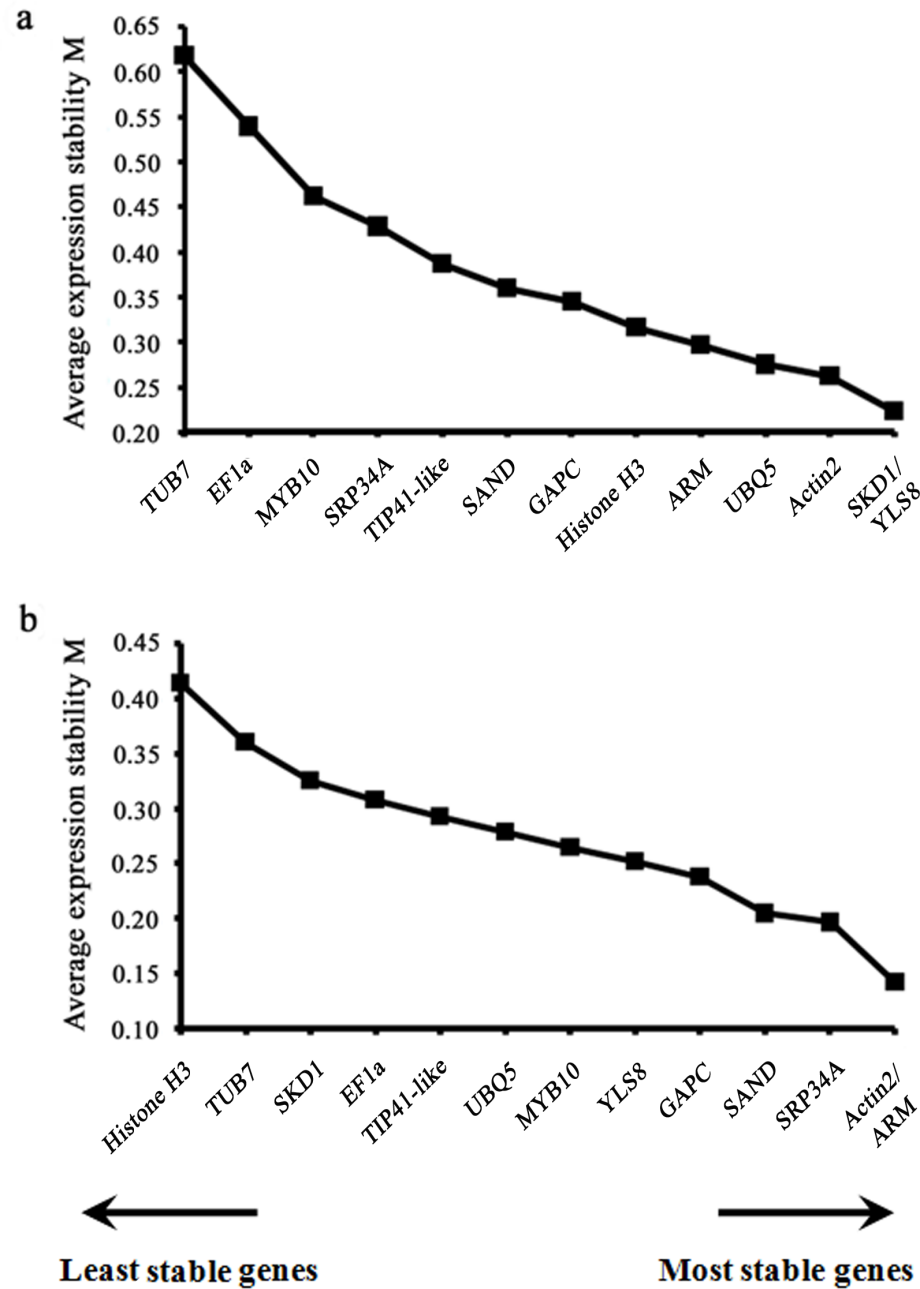
Gene expression stability and ranking of 13 candidate reference genes as calculated by geNorm. Average expression stability (M) of the reference genes was measured during stepwise exclusion of the least stable reference genes. A lower value of average expression stability (M) indicates more stable expression. (**a)** The ‘training_period’ subset; (**b)** the ‘training_space’ subset.

The geNorm tool applied a statistical algorithm to calculate the average expression stability M of all thirteen candidate reference genes. The least stable genes with the highest M values were successively excluded until the most stable genes were determined (Fig 2 and Table 2). All of the tested reference genes showed an overall limited variance, with M values of less than 0.65, which is well below the default limit of 1.5. In the ‘training_space’ subset, the M values of all the candidates were even smaller. The *SKD1* and *YLS8* genes exhibited the lowest variation and highest stability in the ‘training_period’ subset; for the ‘training_space’ subset, the two top ranked candidates were *ARM* and *Actin2*, with an M value of 0.14.

**Table 2:** Ranking of candidate genes according to their expression stability values (M) estimated using the geNorm algorithm.

Rank	training_period		training_space	
	Gene	M	Gene	M
1	*SKD1*	0.22	*ARM*	0.14
2	*YLS8*	0.22	*Actin2*	0.14
3	*Actin2*	0.26	*SRP34A*	0.20
4	*UBQ5*	0.27	*SAND*	0.20
5	*ARM*	0.30	*GAPC*	0.24
6	*Histone H3*	0.32	*YLS8*	0.25
7	*GAPC*	0.34	*MYB10*	0.26
8	*SAND*	0.40	*UBQ5*	0.28
9	*TIP41-like*	0.39	*TIP41-like*	0.29
10	*SRP34A*	0.43	*EF1a*	0.30
11	*MYB10*	0.46	*SKD1*	0.33
12	*EF1a*	0.54	*TUB7*	0.36
13	*TUB7*	0.62	*Histone H3*	0.41
Best combination	*SKD1/ YLS8*		*ARM / Actin2*	

M values computed by geNorm. Stability values are listed from the most stable pair of genes to the least stable.

**Fig 3:**
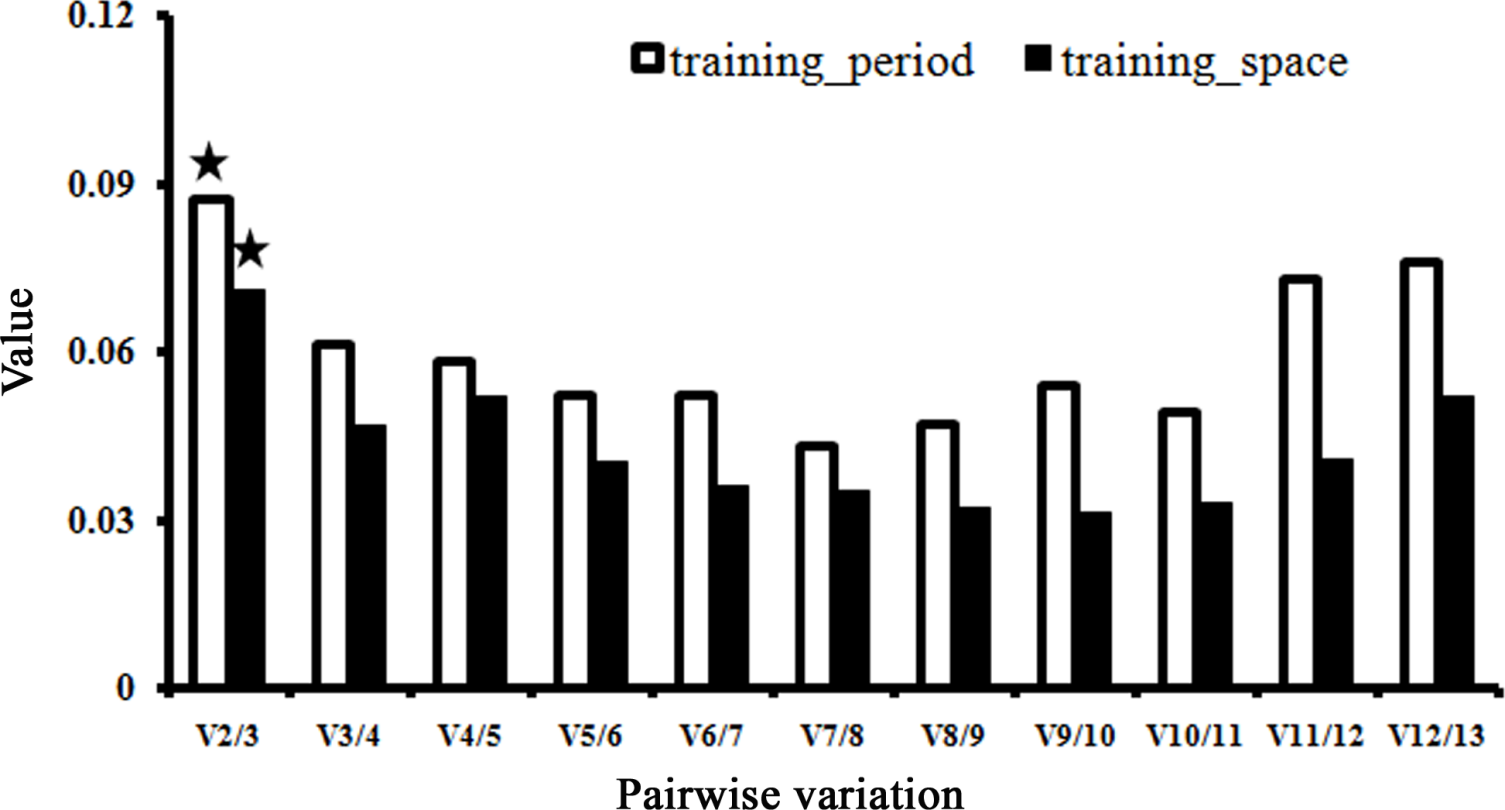
Pairwise variation (V) to determine the optimal number of reference genes for accurate normalization. The pairwise variation (Vn/Vn+1) was analyzed between the normalization factors NFn and NFn+1 by geNorm software. Asterisk indicates the optimal number of genes for normalization.

The optimal number of suitable reference genes for proper normalization was established using pairwise variation values (V). Generally, an additional reference gene was not required until the variation V_n_/V_n+1_ dropped below the given threshold, 0.15. For the two sample subsets, geNorm analysis revealed that the V_2/3_ values were 0.087 and 0.071, respectively (Fig 3), both far below the cut-off of 0.15, indicating that the two most stable reference genes would be sufficient for accurate normalization in each case. Thus, we concluded that *SKD1* and *YLS8* were the optimal multiple reference genes for the ‘training_period’ subset, while *ARM* and *Actin2* were necessary to normalize gene expression for the ‘training_space’ subset.

### Stability assessment of the candidate reference genes by NormFinder

Expression stabilities of the candidate reference genes were also analyzed by NormFinder. All reference gene candidates were ranked based on intra-group variations and converted into a stability value for each candidate. The lowest average stability value for a candidate reference gene indicates the most stable expression. For the ‘training_period’ and ‘training_space’ subsets, *SKD1* and *ARM* were the top ranked candidates, respectively (Table 3).

**Table 3:** Intra-group ranking of the candidate reference genes according to their stability values calculated by NormFinder.

Rank	training_period		training_space	
	Gene	Stab	Gene	Stab
1	*SKD1*	0.065	*ARM*	0.039
2	*Actin2*	0.116	*Actin2*	0.115
3	*YLS8*	0.140	*YLS8*	0.131
4	*Histone H3*	0.195	*SR34A*	0.140
5	*ARM*	0.204	*GAPC*	0.143
6	*TIP41-like*	0.219	*SAND*	0.164
7	*UBQ5*	0.226	*UBQ5*	0.176
8	*GAPC*	0.273	*MYB10*	0.200
9	*SAND*	0.312	*EF1a*	0.210
10	*MYB10*	0.349	*TIP41-like*	0.217
11	*SR34A*	0.398	*SKD1*	0.227
12	*EF1a*	0.556	*TUB7*	0.318
13	*TUB7*	0.673	*Histone H3*	0.466

In addition, inter-group variation was also estimated by NormFinder. For leaf samples in the two subgroups (flat-type trellis system and spindle system) of ‘training_period’, which was referred to as ‘training_period_divided’, the two top ranked candidates were *YLS8* and *SKD1*, which were also the optimal pair (Table 4), in agreement with the results of the distinct statistical algorithm geNorm (Table 2). For the leaf samples in the two subgroups of ‘training_space_divided’, the most stable candidate was *ARM*, and *ARM* and *YLS8* constituted the best combination with a stability value of 0.052 (Table 4).

**Table 4:** Inter-group ranking of the candidate reference genes according to their stability values calculated by NormFinder.

Rank	training_period_divided		training_space_divided	
	Gene	Stab	Gene	Stab
1	*YLS8*	0.030	*ARM*	0.073
2	*SKD1*	0.035	*Actin2*	0.106
3	*Actin2*	0.058	*UBQ5*	0.135
4	*UBQ5*	0.096	*SAND*	0.140
5	*ARM*	0.098	*SR34A*	0.149
6	*Histone H3*	0.105	*GAPC*	0.149
7	*TIP41-like*	0.117	*YLS8*	0.153
8	*GAPC*	0.139	*SKD1*	0.198
9	*SAND*	0.140	*MYB10*	0.216
10	*MYB10*	0.179	*EF1a*	0.222
11	*SR34A*	0.188	*TIP41-like*	0.223
12	*TUB7*	0.286	*TUB7*	0.234
13	*EF1a*	0.299	*Histone H3*	0.389
Best combination	*SKD1 + YLS8*	0.023	*ARM + YLS8*	0.052

### Evaluation of the selected reference genes

**Fig 4:**
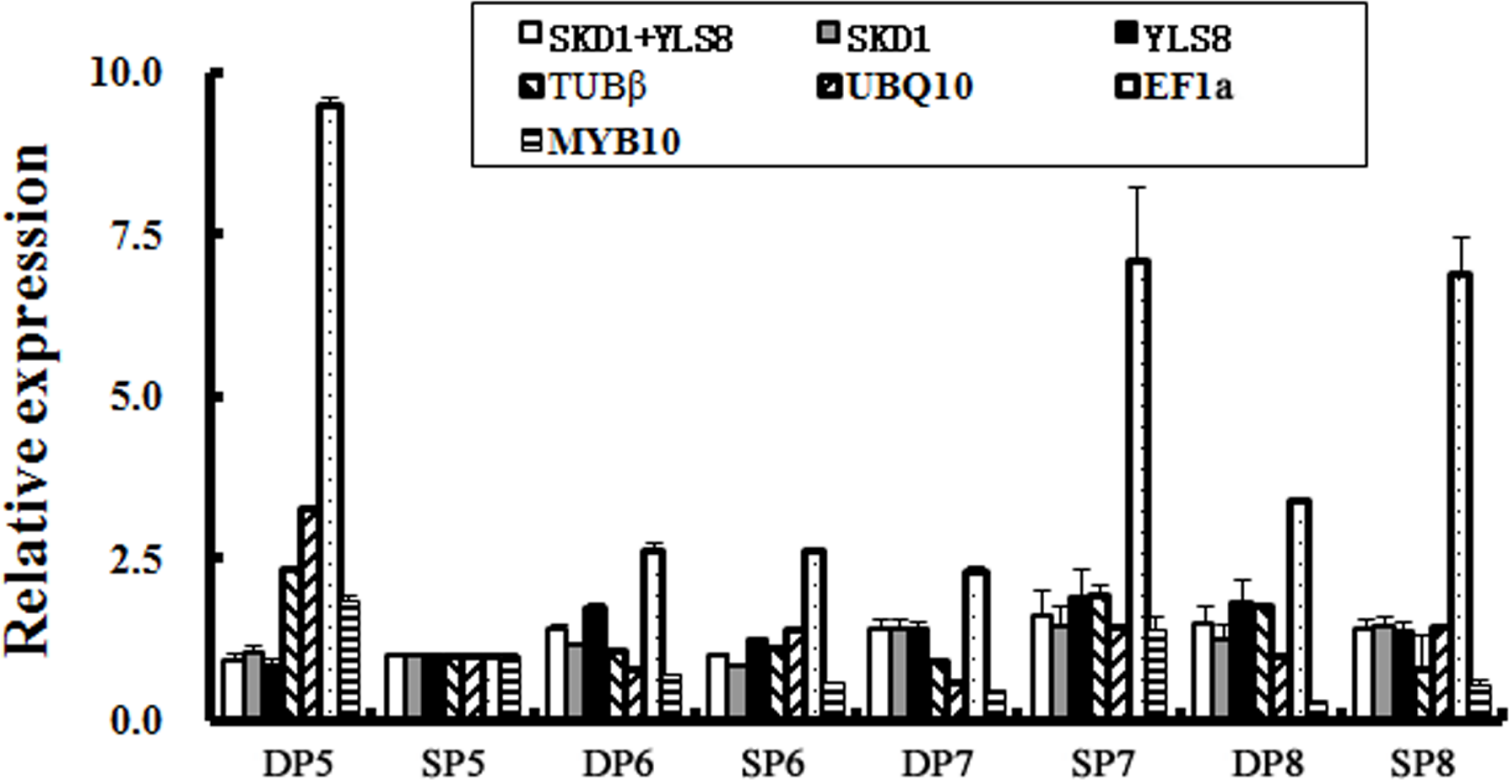
Expression level of *PpsAPX* using validated reference genes for normalization. qRT-PCR data was normalized to the two most stable reference genes (*SKD1* and *YLS8*), two commonly used reference genes (*TUBβ* and *UBQ10*), and two unstable genes (*EF1a* and *MYB10*). *DP*, flat-type trellis system, *SP*, spindle system. The numbers after *DP* and *SP* refer to the month.

To validate the reliability of the selected reference genes, we analyzed the relative expression patterns of the *PpsAPX* gene during leaf development in the two distinct training systems, using the two most stable reference genes and two unstable genes selected by ranking from the two algorithms (geNorm and NormFinder). Two traditional housekeeping genes, *TUBβ* and *UBQ10*, were also used for normalization. As shown in Fig 4, when normalized with *SKD1*, the transcription level of *PpsAPX* was slightly up-regulated during leaf development, reaching its peak in July and then showing steady expression in August. Similar expression patterns were observed when normalized with *YLS8* or in combination with *SKD1* together. On the other hand, a significant over-estimation of the *PpsAPX* expression level could be observed in various leaf development stages when normalized with *EF1a*. For instance, when *PpsAPX* expression was normalized with *EF1a* in sample DP5, its transcription level appeared approximately eight times higher than the estimation obtained using the most stable genes. The relative expression level results were also distorted when we normalized against the other unstable candidate reference genes or classical pear housekeeping genes. These results highlight the importance of the selection of suitable reference genes for reliable results in gene expression.

## Discussion

In pear, the draft genomic sequences of Asiatic pear ‘Suli’ (*Pyrus bretschneideri* Rehd.) and European pear ‘Bartlett’ (*P*. *communis* L.) are now available [42, 43], along with increased transcriptomic resources, which will expedite functional genomics research. Gene expression analysis based on the qRT-PCR technique is a useful research method for increasing our understanding of the interactions between training systems and photosynthetic efficiency-related gene function. Reliable quantification results through qRT-PCR are largely dependent on the high expression stability of endogenous reference genes [23, 44]. However, there is no ideal reference gene that could be stably expressed in all situations [45–47]. Thus, it is advisable to systematically validate the reference genes prior to their use in each specific experimental situation.

In this study, four novel candidate reference genes retrieved from mRNA high-throughput sequencing data (S1 Table) and nine common reference genes with highly stable expression levels under other tested experimental conditions in pear [37–39] were selected to determine the most reliable gene(s) for accurate normalization, instead of using housekeeping genes chosen arbitrarily. To the best of our knowledge, the present work is the first systematic evaluation of the expression stability of reference genes in training system studies. We have employed two distinct software packages, geNorm and NormFinder, to evaluate the expression stability of the candidates in two independent experimental subsets (Tables 2–4 and Fig 2). GeNorm uses a pairwise approach to select the two genes with the least variation in their expression ratios, while NormFinder uses a model-based variance estimation approach to identify candidate reference genes with lowest intra- and inter-group variation [31, 32]. Owing to their distinct statistical algorithms, the assessment results obtained by distinct statistical algorithms were not identical. It is noteworthy that both programs yielded similar overall ranking orders with subtle variation under certain experimental conditions. The candidates in the last two positions, *EF1a*/*TUB7* for ‘training_period’ and *TUB7*/*Histone H3* for ‘training_space’, were consistently predicted by both algorithms, indicating that these genes were inadequate for transcript normalization in pear under these experimental conditions. For ‘training_period’, *SKD1* and *YLS8* were ranked as the most stable single reference genes and the best combination by both programs, no matter whether intra- or inter-group variation was considered. For ‘training_space’, both algorithms recommended *ARM* and *Actin2* as the top two most stable reference genes, but the best combination of genes varied slightly: geNorm selected *ARM*+*Actin2* as the optimal pair, while the best combination was *ARM*+*YLS8* according to the NormFinder inter-group variation analysis. Some researchers preferred the NormFinder algorithm owing to its robust design and ability to provide more reliable information on the stability of a single reference gene [44, 48]. We found that NormFinder affirmed the optimal single reference gene determined by geNorm. Here, we counted on the result of geNorm, since it systematically analyzes optimal multiple reference genes. Thereby, *SKD1* (or *YLS8*) and *ARM* were the most stable single reference genes for the ‘training_period’ and ‘training_space’ subsets, respectively, but their validity may still lower than the optimal pairs *SKD1*+*YLS8* and *ARM*+*YLS8* as assessed by geNorm, based on the hypothesis that a single reference gene is less stable than a combination of multiple reference genes [26, 49]. The current work is the first to propose *SKD1* and *ARM* sequences as stable reference genes. The homolog of *SKD1* in *Arabidopsis thaliana* contributes to vacuolar trafficking and maintenance of the large central vacuole of plant cells [50, 51], in agreement with its stable expression in pear because it is probably involved in basic processes of cell maintenance. The sequence of *ARM* had a low similarity (E-value = 0.28) to the *Arabidopsis* gene (Table 1) and no GO or KEGG annotation in our previous transcriptome analysis. While we can not speculate arbitrarily on its molecular function and biological process, this novel candidate gene is a good choice for normalization in gene expression studies of pear in distinct training systems.

Both software packages indicated that *GAPC*, *SAND*, *TIP41-like*, *MYB10* and *SRP34* ranked in the middle positions among the subsets studied. *GAPC* was considered the most appropriate reference gene in pear when considering fruit tissues or phytohormone treatments [37]. However, our results revealed that the expression stability of other two novel genes, S*KD1* and *ARM*, was superior to that of *GAPC* during leaf development in distinct training systems (Tables 2–4). Similar results were observed in *Petunia*; *GAPDH* was considered the gene least stably expressed when assessed during leaf and flower development [52]. Based on NormFinder stability values in pear flower organ samples, *SAND* was the top reference gene, and *TIP41-like* was a variable gene [38]. In contrast, using the same algorithm, our results showed that the expression stability of *TIP41-like* was higher than that of *SAND* in the ‘training_period’ subset, although neither was the most stable reference gene (Tables 3 and 4). Furthermore, it is interesting to note that the two other novel candidates, *MYB10* and *SRP34A*, had approximately equal expression levels between fruit libraries in these distinct training systems by high-throughput sequencing (S2 Table). However, we found that the expression of these two genes varied among the 14 leaf samples in the same training systems, ranking in the middle position with Ct values spanning 24.75–26.86 and 23.62–25.43, respectively. This outcome indicates that the expression of these two candidates was affected by tissue type. These results highlight the necessity to assess suitable reference genes for each experimental condition, and not to assume that reference genes that have stable expression in one experimental condition are optimal in another condition, even in the same species.

The suitability of the selected reference genes has been validated by analyzing the expression profile of a *PpsAPX* gene encoding a key enzyme that plays a central role in scavenging H_2_O_2_ under stress conditions [53]. Similar expression patterns of *PpsAPX* were described when the stable single references *SKD1* and *YLS8* and the optimal reference pair *SKD1*+*YLS8* were employed (Fig 4). We found that the expression of *PpsAPX* was slightly increased during early leaf development and then stably expressed duing fruit harvesting period (July and August), regardless of whether the flat-type trellis system or traditional spindle system was considered. The expression analysis results were consistent with previous research ideas that leaf senescence are associated with the formation of free radicals but that this effect can be retarded by removing reproductive tissues (flower or fruit) [54]. As expected, notably conflicting expression differences were estimated with the two unstable references or with two commonly used reference genes compared to the results normalized by the stable candidate genes, leading to too high (or relatively low) misinterpretation of the *PpsAPX* expression level. These results further proved the reliability of the optimal single and double reference gene(s) selected by geNorm and NormFinder in our study.

## Conclusions

This is the first study to comprehensively analyze the stability of a set of reference genes in distinct training systems. Two novel candidates, *SKD1* and *ARM*, display high stability in the ‘training_period’ and ‘training_space’ subsets, respectively. Reference pairs *SKD1*+*YLS8* and *ARM*+*Actin2* are the best multiple reference genes for the two subsets mentioned above. In addition, we also showed that some commonly used reference genes, such as *TUBβ* and *UBQ10*, could be inadequate for transcript normalization under particular experimental conditions in pear. Our results provide a foundation for extensive use of qRT-PCR in gene expression analysis for future training system studies in pear, which could be beneficial for post-genomic research and revealing the underlying molecular mechanism of the effects of training systems on leaf photosynthesis and fruit quality.

## Supporting information

S1 Fig The two training systems used in this study. The flat-type trellis system (Double Primary Branches Along the Row) has two primary branches bent in opposite directions along the row and hung naturally on the two horizontal wires. In spring, the branches of the trees had many new leaves and flowers on them (a). Lateral branches with high-quality fruit were evenly distributed on the primary branches in summer (b), but the leaves gradually fell in autumn (c). In the spindle system, the tree has a central and vertical leader, with a few horizontal branches that are shorter at the tree top than at the bottom (d). The schematic illustration (aerial view) of leaf samples collection for the ‘training_space’ subset (e). Samples were collected from exterior, central and interior parts of the trees in that flat-type trellis system (upper part) and spindle system (bottom part). Interior parts (I), central part (C), and exterior part (E) were approximately 0–0.5 m, 0.5–1.0 m and more than 1.0 m away from the trunk.
(TIF)

S2 Fig The RNA quality of 14 samples assessed by Agilent 2100 Bioanalyzer and agarose gel showing amplification of PCR product of the expected amplicon size for each gene tested in this study. a. Gel-like image produced by Agilent 2100 bioanalyzer showing the results of separate bands of RNA samples. A typical electropherogram of leaf RNA include clearly visible 28/18S rRNA peak. Two additional prominent peaks migrated faster than cytoplasmic 18S rRNA, indicating an abnormal migration of 16S and 23S rRNA from chloroplasts, presumably due to secondary structures effects that were not resolved by non-denaturing capillary electrophoresis. [FU] represent fluorescence. DP, flat-type trellis system, SP, spindle system. The numbers after DP and SP refer to the month. DP/SP-x was the DP or SP leaf samples collected from exterior, central and interior parts of the trees, x = In, Ce and Ex represented Interior parts, central part, and exterior part. b. Equal amounts of cDNA from all tested samples were mixed as the templates. The thirteen amplicon sizes were verified with 1.2% agarose gel electrophoresis and ethidium bromide staining (b). M, P, T and R represent DNA size marker, PCR for positive amplifications, no-template control and no-reverse-transcriptase control.
(TIF)

S3 Fig Dissociation curve data for the 13 reference genes tested. (TIF)

S1 Table Four novel candidate reference genes retrieved from mRNA high-throughput sequencing data. Transcripts were retrieved from high-throughput sequencing results of fruit tissues between the flat-type trellis system and traditional spindle system.
(DOCX)

S2 Table Sequence information of the 13 candidate reference gene amplicons. (DOCX)
