# Supplementary figures and images for: Selection and validation of suitable reference genes for qRT-PCR analysis in pear leaf tissues under distinct training systems
Source: PLoS One. 2018 Aug 23;13(8):e0202472. doi: 10.1371/journal.pone.0202472 (PMC6107188; doi:10.1371/journal.pone.0202472)

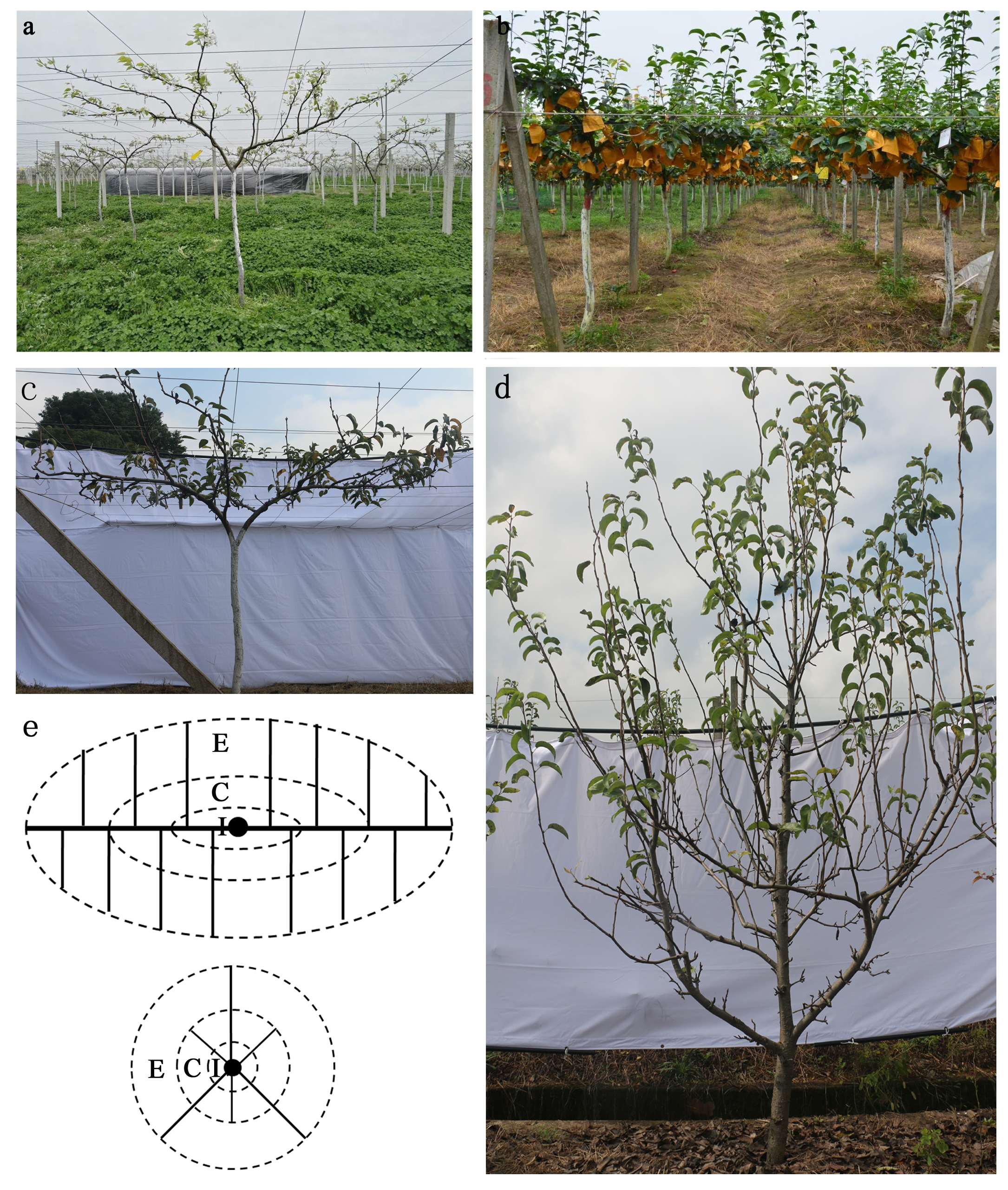

Supplement: S1 Fig — The flat-type trellis system (Double Primary Branches Along the Row) has two primary branches bent in opposite directions along the row and hung naturally on the two horizontal wires. In spring, the branches of the trees had many new leaves and flowers on them (a). Lateral branches with high-quality fruit were evenly distributed on the primary branches in summer (b), but the leaves gradually fell in autumn (c). In the spindle system, the tree has a central and vertical leader, with a few horizontal branches that are shorter at the tree top than at the bottom (d). The schematic illustration (aerial view) of leaf samples collection for the ‘training_space’ subset (e). Samples were collected from exterior, central and interior parts of the trees in that flat-type trellis system (upper part) and spindle system (bottom part). Interior parts (I), central part (C), and exterior part (E) were approximately 0–0.5 m, 0.5–1.0 m and more than 1.0 m away from the trunk. (TIF) [file pone.0202472.s001.tif]

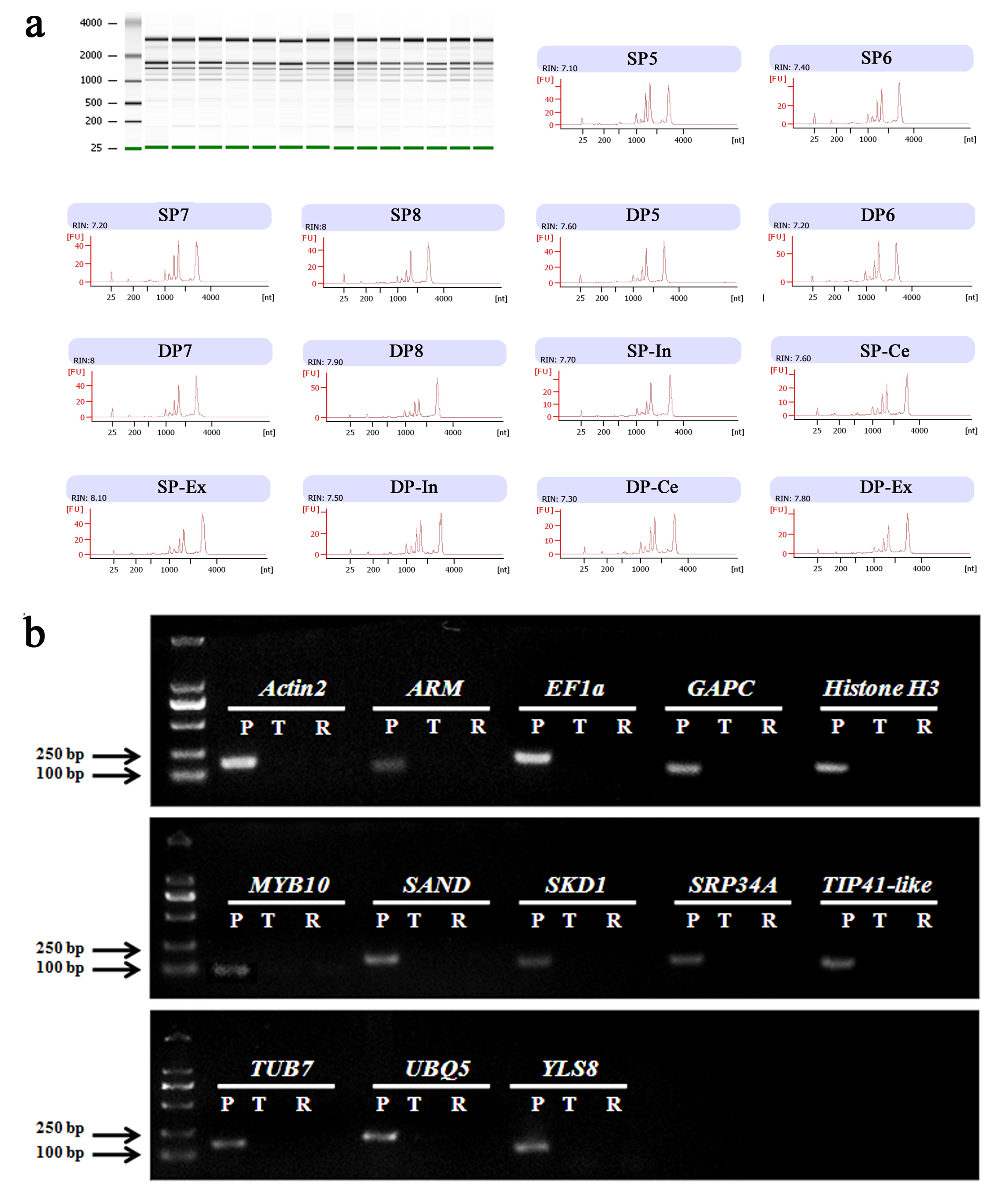

Supplement: S2 Fig — a. Gel-like image produced by Agilent 2100 bioanalyzer showing the results of separate bands of RNA samples. A typical electropherogram of leaf RNA include clearly visible 28/18S rRNA peak. Two additional prominent peaks migrated faster than cytoplasmic 18S rRNA, indicating an abnormal migration of 16S and 23S rRNA from chloroplasts, presumably due to secondary structures effects that were not resolved by non-denaturing capillary electrophoresis. [FU] represent fluorescence. DP, flat-type trellis system, SP, spindle system. The numbers after DP and SP refer to the month. DP/SP-x was the DP or SP leaf samples collected from exterior, central and interior parts of the trees, x = In, Ce and Ex represented Interior parts, central part, and exterior part. b. Equal amounts of cDNA from all tested samples were mixed as the templates. The thirteen amplicon sizes were verified with 1.2% agarose gel electrophoresis and ethidium bromide staining (b). M, P, T and R represent DNA size marker, PCR for positive amplifications, no-template control and no-reverse-transcriptase control. (TIF) [file pone.0202472.s002.tif]

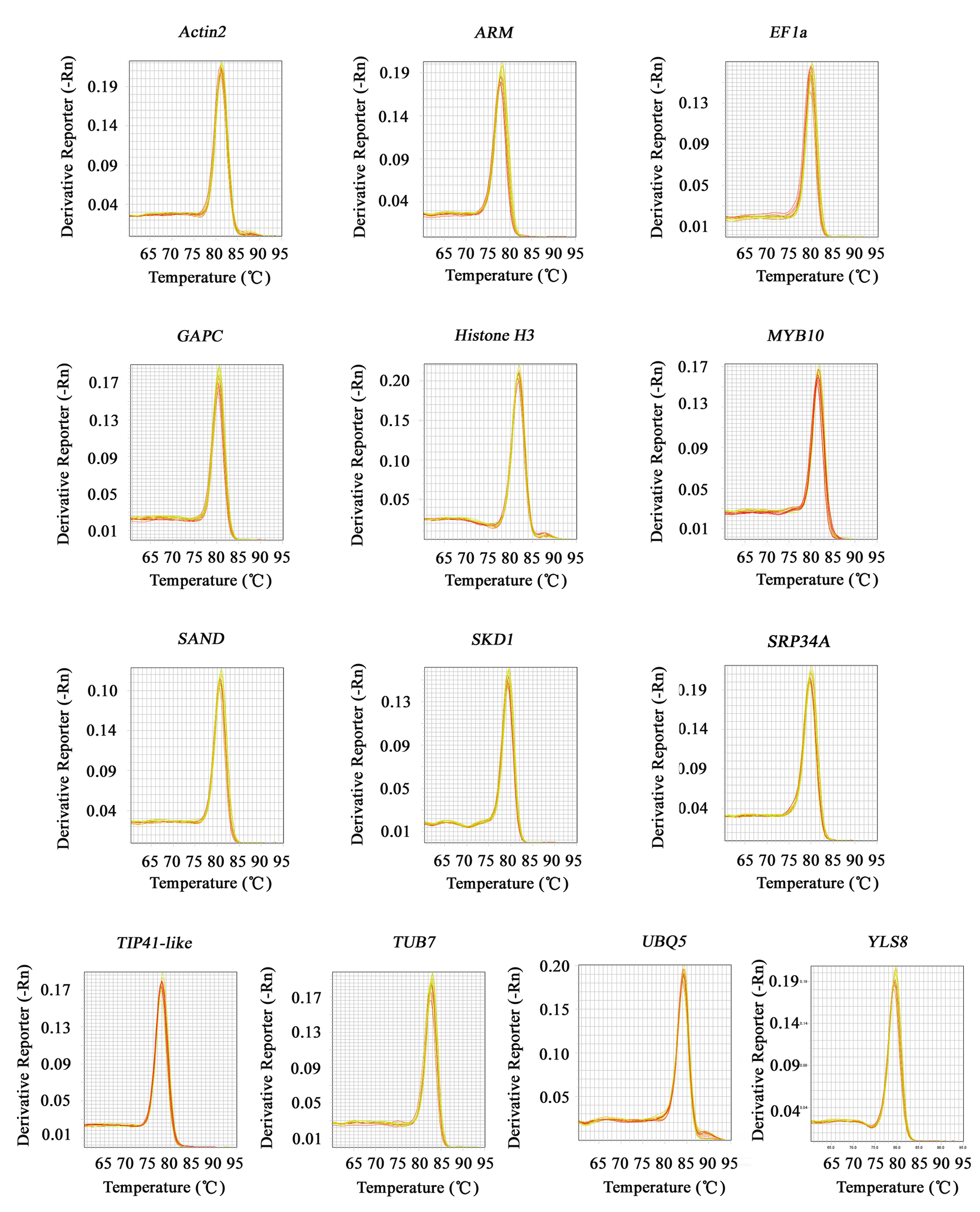

Supplement: S3 Fig — (TIF) [file pone.0202472.s003.tif]
