# Supplementary material for: Selection and validation of suitable reference genes for qRT-PCR analysis in pear leaf tissues under distinct training systems
Source: PLoS One. 2018 Aug 23;13(8):e0202472. doi: 10.1371/journal.pone.0202472 (PMC6107188; doi:10.1371/journal.pone.0202472)
Supplement: S1 Table — Transcripts were retrieved from high-throughput sequencing results of fruit tissues between the flat-type trellis system and traditional spindle system. (DOCX) [file pone.0202472.s004.docx]

**S1 Table. Four novel candidate reference genes retrieved from mRNA high-throughput sequencing data.**

| **Gene name** | **GeneID** | **log2(Fold change)** | **p-value** | **q-value** |
| --- | --- | --- | --- | --- |
| *ARM* | rna33411 | 0.419 | 0.257 | 0.016 |
| *MYB10* | rna29837 | 0.164 | 0.023 | 0.055 |
| *SKD1* | MSTRG.11298.4 | 0.132 | 0.059 | 0.079 |
| *SRP34A* | rna5849 | 0.17 | 0.025 | 0.056 |
