# Supplementary material for: Selection and validation of suitable reference genes for qRT-PCR analysis in pear leaf tissues under distinct training systems
Source: PLoS One. 2018 Aug 23;13(8):e0202472. doi: 10.1371/journal.pone.0202472 (PMC6107188; doi:10.1371/journal.pone.0202472)
Supplement: S2 Table — (DOCX) [file pone.0202472.s005.docx]

**S2 Table. Sequence information of the thirteen candidate reference genes amplicons.**

| **Gene abbreviation** | **Candidate reference genes amplicons** |
| --- | --- |
| *Actin2* | CTCCCAGGGCTGTGTTTCCTAGTATTGTTGGTCGCCCACGACACACAGGTGTCATGGTTGGTATGGGTCAGAAGGATGCCTATGTAGGTGATGAAGCACAGTCGAAAAGAGGTATCCTTACCTTGAAGTATCCCATTGAGCACGGTATAGTGAGCAACTGGGATGACATGGAG |
| *ARM* | CAAGGGCATTCTTTCGGAGCAACTGGTTTCTGTGAAAGAAGAAAGCATGAGAATATTGAAGGACTTCATCACCAGACACAATGTTCCTAATGATGTCCCTG |
| *EF1a* | GGTGTGAAGCAGATGATTTGCTGCTGTAACAAGATGGATGCCACCACTCCTAAGTACTCTAAGGCAAGGTATGAAGAAATCGTGAAGGAAGTTTCTTCCTACCTGAAGAAGGTTGGGTACAACCCCGACAAAATCGCCTTTGTTCCCATATCTGGGTTTGAGGGTGA |
| *GAPC* | TGGTGTGAACGAGAAGGAATACAAGCCAGACATTCACATTCTTTCCAATGCCAGTTGCACTACCAACTGCCTTGCTCCCCTTGCCAAGGTTATCAACGACAGGTTTGGGATTGTTGAGGG |
| *Histone H3* | GTCAAGAAGCCCCACAGATACCGCCCTGGTACTGTCGCTCTTCGTGAAATCCGTAAGTACCAGAAGAGTACTGAGCTCTTGATCAGGAAGTTGCCATTCCAGAGGCTTGTTCGTGAAATTGCCCAGGATTTCAAGACTGATCTGCGTTTCCAG |
| *MYB10* | GGGAACAACAGCAAACGATCGTCGTCTTCCGAGGACAAGCCACCGTCGCCGAAACGACCGAAGGTGGATAATGGTAGTGCTTCGGAG |
| *SAND* | CCCAGGACTTTGAGCTTTATGCAGCTTTTGATCCACTTGCAGACAAGGCATTGGCAATAAAGACTTGCAACCGGGTTTGTCAGTGGGTGAAAGATGTGGAAAACGAGATTTTTTTGCTAGGAGCAAGCCCCTTTTCATGGTGATA |
| *SKD1* | CTTCCGCCTCCTATCACAAAAACAGATTTCGATAAGGTTCTTGCTAGACAGAGGCCTACAGTGAGCAAAAGTGACCTTGAAGTCCATGAGAGATTCACAAGAGAGTTCGGAGAGGAAGGGTGATGAA |
| *SRP34A* | CGGTAGTGCCTGATTCTCTGGCAAGTCATCCCCGCGGCGTTCTTGGGTGTTAGATCCGCGAAGATTCATCATATCCGAGTGAACTTGTTGAAAAATTTCGTTTTTAAGAACCTTGGTTATCTTTGTTATGGCGAGTATG |
| *TIP41-like* | ATCCAAGCATCATCAGCCAAAGGCTTCCTGTGATCATGCATAAGACCCAAAAGCTAAAGGTACCTGGTAATATGTAAGGTTGTAAATTGTCTCCCTGCAAGAGTTATTGTTCC |
| *TUB7* | TGGGCTTTGCTCCTCTTACTTCACGTGGCTCTCAGCAGTACCGGGCCCTGACTGTTCCAGAGCTCACACAGCAAATGTGGGACTCCAAGAACATGATGTGTGCTGCTGATCCCCGACATGGACGTTACCTAACAGCATCAGCCATGTTCCGTGGTAAGATGAGCACGAAGG |
| *UBQ5* | ACCCTCGCCGACTACAACATCCAGAAGGAGTCCACCCTACACCTTGTGCTCCGCCTCCGCGGTGGCGCCAAGAAGAGGAAGAAGAAGACCTACACCAAGCCCAAGAAGATCAAGCACAAGCACAAGAAGGTGAAGCTCGCAGTGCTCCAGTTCTACAAGGTGGATGACTCCGGCAAGGTCCAGAGGCTGCGGAAGGAGT |
| *YLS8* | TGAGGTGCTGGCTTCTGTTGCTGAGACAATAAAAAACTTTGCTGTGATTTACCTCGTGGACATCACGGAGGTTCCTGATTTCAACACAATGTATGAGCTCTACGATCCATCAACGGTCA |
